# Kenya’s emergency-hire nursing programme: a pilot evaluation of health service delivery in two districts

**DOI:** 10.1186/1478-4491-12-16

**Published:** 2014-03-17

**Authors:** Stephen M Vindigni, Patricia L Riley, Francis Kimani, Rankesh Willy, Patrick Warutere, Jennifer F Sabatier, Rose Kiriinya, Michael Friedman, Martin Osumba, Agnes N Waudo, Chris Rakuom, Martha Rogers

**Affiliations:** 1 University of Washington School of Medicine, 1959 NE Pacific Street, Box 356421, Seattle, WA 98195-6421, USA; 2 Division of Global HIV/AIDS, Center for Global Health, Centers for Disease Control and Prevention, 1600 Clifton Rd, Atlanta, GA 30333, USA; 3 Office of the Director of Medical Services, Kenya Ministry of Medical Services, Afya House, Cathedral Road, P.O. Box 30016, Nairobi, Kenya; 4 Office of the Chief Nursing Officer, Kenya Ministry of Medical Services, Afya House, Cathedral Road, P.O. Box 30016, Nairobi, Kenya; 5 Office of Health Management Information Systems, Ministry of Medical Services, Afya House, Cathedral Road, P.O. Box 30016, Nairobi, Kenya; 6 Kenya Health Workforce Project, Lower Hill Duplex, P.O. Box 7808–00200, Nairobi, Kenya; 7 Lillian Carter Center for International Nursing, Nell Hodgson Woodruff School of Nursing, Emory University, 1520 Clifton Road, Atlanta, GA 30322-4201, USA

**Keywords:** Emergency-hire programme, Human resources for health, Human resource information systems, Health management information systems, Kenya, Nursing, Primary care

## Abstract

**Objective:**

To assess the feasibility of utilizing a small-scale, low-cost, pilot evaluation in assessing the short-term impact of Kenya’s emergency-hire nursing programme (EHP) on the delivery of health services (outpatient visits and maternal-child health indicators) in two underserved health districts with high HIV/AIDS prevalence.

**Methods:**

Six primary outcomes were assessed through the collection of data from facility-level health management forms—total general outpatient visits, vaginal deliveries, caesarean sections, antenatal care (ANC) attendance, ANC clients tested for HIV, and deliveries to HIV-positive women. Data on outcome measures were assessed both pre-and post-emergency-hire nurse placement. Informal discussions were also conducted to obtain supporting qualitative data.

**Findings:**

The majority of EHP nurses were placed in Suba (15.5%) and Siaya (13%) districts. At the time of the intervention, we describe an increase in total general outpatient visits, vaginal deliveries and caesarean sections within both districts. Similar significant increases were seen with ANC attendance and deliveries to HIV-positive women. Despite increases in the quantity of health services immediately following nurse placement, these levels were often not sustained. We identify several factors that challenge the long-term sustainability of these staffing enhancements.

**Conclusions:**

There are multiple factors beyond increasing the supply of nurses that affect the delivery of health services. We believe this pilot evaluation sets the foundation for future, larger and more comprehensive studies further elaborating on the interface between interventions to alleviate nursing shortages and promote enhanced health service delivery. We also stress the importance of strong national and local relationships in conducting future studies.

## Background

Despite having 25% of the global disease burden, sub-Saharan Africa has only 3% of the world’s health workers [1,2]. The etiology of these shortages includes retirement, pre-service training costs, out-migration, and the effects of HIV/AIDS [3-6]. Each factor challenges a country’s ability to maintain health-care worker density.

Related to this scarcity of workers, health systems researchers have documented that workforce density inversely correlates with mortality rates of vulnerable populations. Staffing levels have also been associated with the provision of essential primary care services [7-9]. Although studies are few, researchers agree that improving human resources with skilled providers translates into improved primary health care [10,11]. Chen postulates that the critical minimum health-care worker coverage threshold is 2.5 workers per 1000 population, which corresponds to an 80% coverage for two health service indicators—skilled birth attendance and measles vaccination coverage [7]. When the provider to population ratio falls below this level, health outcomes and preventive health services are compromised. At current health-care worker levels, most sub-Saharan countries are unable to meet these needs, particularly countries with sizable HIV/AIDS populations. In 2012, Kenya had an overall HIV prevalence of 5.6% a decrease from 7.2% in 2007 with Nyanza Province significantly higher at 14.9% in 2007 and 15.1% in 2012 [12]. While host countries and donor organizations have begun to address provider shortages with investments in pre-service training, resolving the provider imbalance in these settings will require years of sustained support [13].

In recognition of the shortage, the Government of Kenya (GoK) declared health worker staffing a major challenge to health development [14]. As such, improving human resources for health (HRH) has become a priority for the Kenya Ministry of Health (MOH) and is now an integral feature of donor funding guidelines [6].

Although there are varied approaches for alleviating nursing shortages in remote and underserved areas, Kenya’s response was to initiate an Emergency-Hire Programme (EHP) in 2005. This programme comprises a fast-track hiring and deployment intervention supported by multiple donor organizations [15]. The EHP’s intent was to scale-up health-care providers, focusing on nursing staff within the public sector. Most EHP nurses were hired on one- to three-year contracts with the goal of personnel absorption into Kenya’s civil service by the end of their contract [15,16].

To date, minimal research has evaluated the effects of Kenya’s EHP intervention on the delivery of health-care services. Although Gross demonstrated short-term increases in nursing staff, no studies have evaluated the effect on core health services at the local level, nor the feasibility of conducting such a study [15,16].

While we acknowledge the multiple approaches to addressing the nursing shortage, one of which is the creation of an EHP, the purpose for this evaluation is not to comment on the merits of this specific intervention, its implementation, or whether an alternative hiring programme would have been more effective. Adequately responding to Kenya’s shortage requires a multifaceted approach taking into account pre-service training, retention strategies, alleviation of financial constraints, and adequate working conditions. We hypothesize that an increase in the provision of nurses to rural, underserved health facilities results in increased delivery of health services; however, this study’s main objective was to assess the feasibility of utilizing a small-scale, low-cost pilot approach to evaluate the delivery of these services. We aim to show the short-term impact of the EHP with regard to increasing health-care services at local sites and evaluate the effect of increased staffing on health service delivery (outpatient visits and maternal-child health indicators) in two underserved health districts with high HIV/AIDS prevalence. Given the small scale of this study, there are inherent limitations including small sample size, lack of controls, and limited data availability; these barriers are discussed in more detail below.

## Methods

This evaluation engaged both qualitative and quantitative methodologies aimed at assessing the feasibility of a low-cost, small-scale, pilot study to assess the impact of the EHP on health service delivery. With fieldwork conducted over two months and data collection from 2004 to 2010, the evaluation focused on the provision of services in 13 health facilities that benefitted from the hiring of EHP nurses.

**Figure 1:**
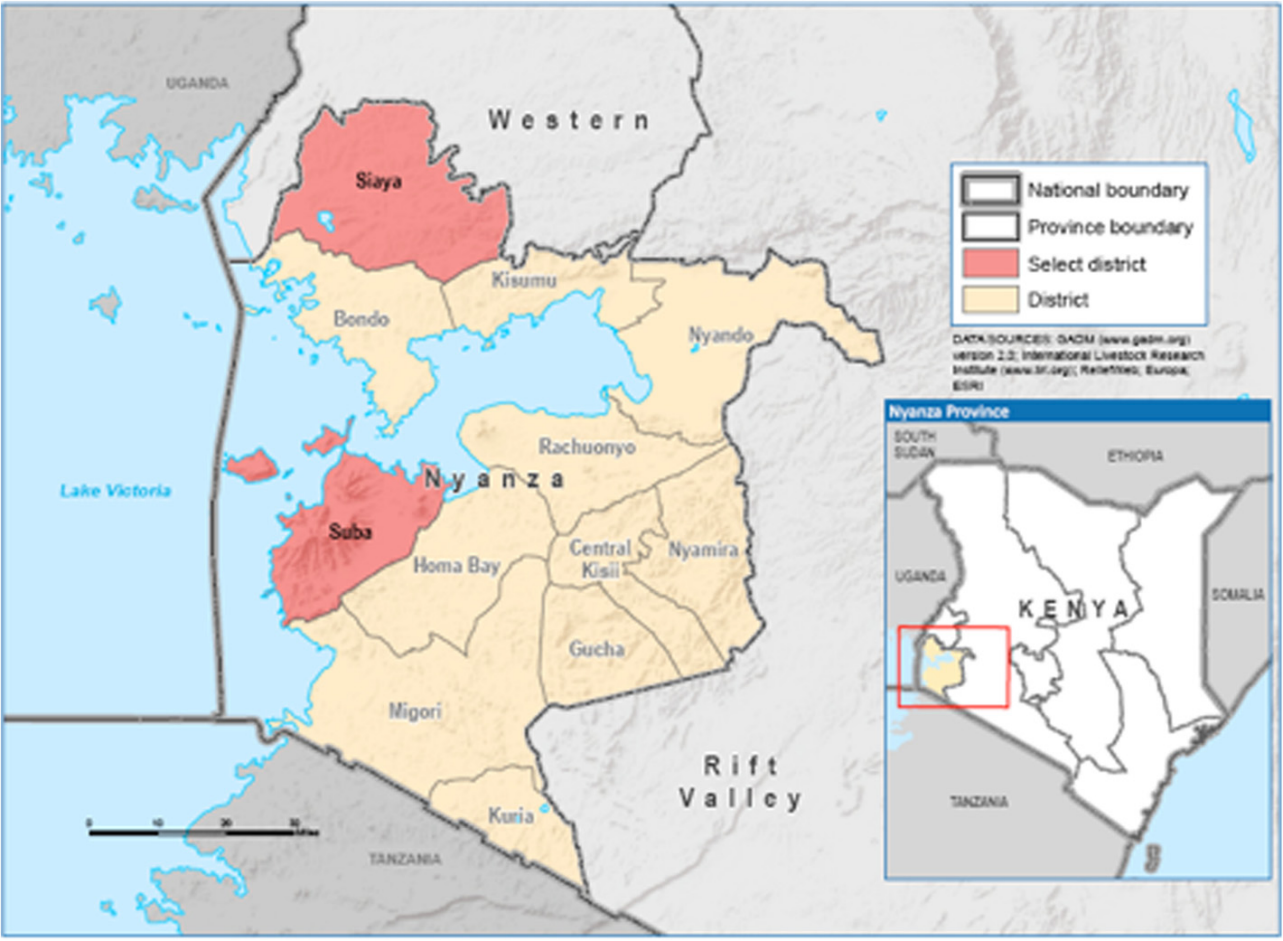
Map of Kenya and Nyanza Province.

We performed an ecological analysis by comparing the level of health services before and after the assignment of EHP nurses within facilities in two MOH districts. Since most nurses were hired in 2007, this year was used as the referent, or intervention year. The two districts with the greatest quantity of EHP nurses were selected for field-level evaluation—Suba and Siaya Districts, both within Nyanza Province—where HIV prevalence is greatest (Figure 1). The primary investigator travelled with two Kenyan health officers to 13 health facilities comprising a combination of district hospitals, sub-district hospitals, and health centers during May and June 2010. These facilities were selected based on increases in staffing levels, geographic convenience, and to obtain a variety of facility types.

Information on health-care workers, including EHP nurses, was obtained from Kenya’s Human Resource Information System (HRIS), known as the Kenya Health Workforce Information System (KHWIS), which consists of two databases—one housing information on the regulation of nurses and the other containing information on nurse deployment [3]. De-identified deployment data on nurses assigned to each district (MOH and emergency-hire) were obtained using the KHWIS. Each cohort was analysed individually and in total and stratified by province, district, and individual health facility. Nursing data were analysed for demographics, training level, and health facility assignment. Microsoft Access (Microsoft, Redmond, WA, USA) and Stata (StataCorp, College Station, TX, USA) were used for statistical analysis.

Provision of health services was determined using Kenya’s HMIS, which collects data at individual health facilities. This information is aggregated and submitted to the MOH’s national office. The HMIS standardized forms document patient services, such as, reproductive health, immunization coverage and other disease surveillance and health outcome parameters (forms in Additional file 1). Health service delivery data was obtained from each health facility visited within Siaya and Suba Districts. Previously collected, monthly-aggregated HMIS data was abstracted dating back to January 2004 through May 2010, although 2004–2005 data was significantly incomplete, therefore, it was not included in this evaluation. A listing of health services is described in Table 1. Abstracted data was analysed in aggregate and stratified by district and facility. Data were averaged based on the quantity of data points available to account for unavailable data.

**Table 1:** Results of aggregate annual data in Siaya and Suba Districts

	**District**	**Rate ratio**			
		**2006**	**2007**	**2008**	**2009**
Total general outpatients	Siaya	0.50**	Ref	1.02	0.98
	Suba	0.54*	Ref	1.08	0.91
Total vaginal deliveries	Siaya	0.50****	Ref	1.10	1.02
	Suba	0.54****	Ref	1.06	1.02
Total caesarean sections	Siaya	0.37****	Ref	1.21*	2.36****
	Suba	0.10****	Ref	0.28****	1.43****
Total antenatal care (ANC) attendance	Siaya	0.64*	Ref	1.06	0.81
	Suba	0.50****	Ref	0.70	0.63**
Total ANC clients tested for HIV	Siaya	0.21****	Ref	0.89	0.75*
	Suba	0.28****	Ref	0.90	0.71**
Total deliveries to HIV-positive women	Siaya	0.19***	Ref	1.28*	1.35*
	Suba	0.46****	Ref	1.52	2.10****

*significant at 0.05.

**significant at 0.01.

***significant at 0.001.

****significant at 0.0001.

One key informant interview was also conducted with a convenience sample of medical, nursing, and clinical officer managers at each site (n = 13) to provide further insight into the quantitative trends. Topics for discussion included EHP service delivery benefits, existing challenges regarding service provision, and ideas for increasing the quantity and quality of primary care services. Efforts were made to identify additional causes of service delivery change beyond the deployment of nursing staff (e.g. medical supply availability, medical/public health programmes and campaigns, environmental factors). Face-to-face, 30- to 45-minute discussions were facilitated by the primary author (SV), a male, public health professional with no prior relationship to the discussants. Although the interviews were structured with the same questions at each site (interview guide and questions in Additional file 2), these were intended to be informal discussions without audio-video recording. No staff member refused participation and the only other persons present were two co-authors.

To evaluate changes in services in association with EHP implementation, we focused on six indicators—total general outpatients, vaginal deliveries, caesarean sections, antenatal care (ANC) attendance, ANC clients tested for HIV, and deliveries from HIV-positive women. Each of these indicators was collected independently and summed by facility for each year between 2006 and 2009. Annual facility averages were adjusted based on the number of months of data available. Data obtained prior to 2006 were not included since monthly HMIS records were incomplete for those years.

Since the EHP was mainly implemented in 2007, the indicators were analysed for changes in sums for each year compared to 2007. A Poisson regression model was used with a year as a categorical predictor, and year 2007 as the referent/intervention category. To account for correlation of data within sites, we specified the working correlation matrix as exchangeable, which assumes the same correlation between any two elements of a cluster and is a reasonable assumption for these data. In this way, we obtained rate ratios and 95% confidence intervals, which assess the rate of the indicator for each year compared to 2007. If the rate ratio is greater than one, this implies the rate of the indicator was greater in that year compared to 2007. If the rate ratio is less than one, it indicates the rate was less in that year compared to 2007. Finally, if the rate ratio is exactly one, the rate was the same compared to 2007.

## Results

**Figure 2:**
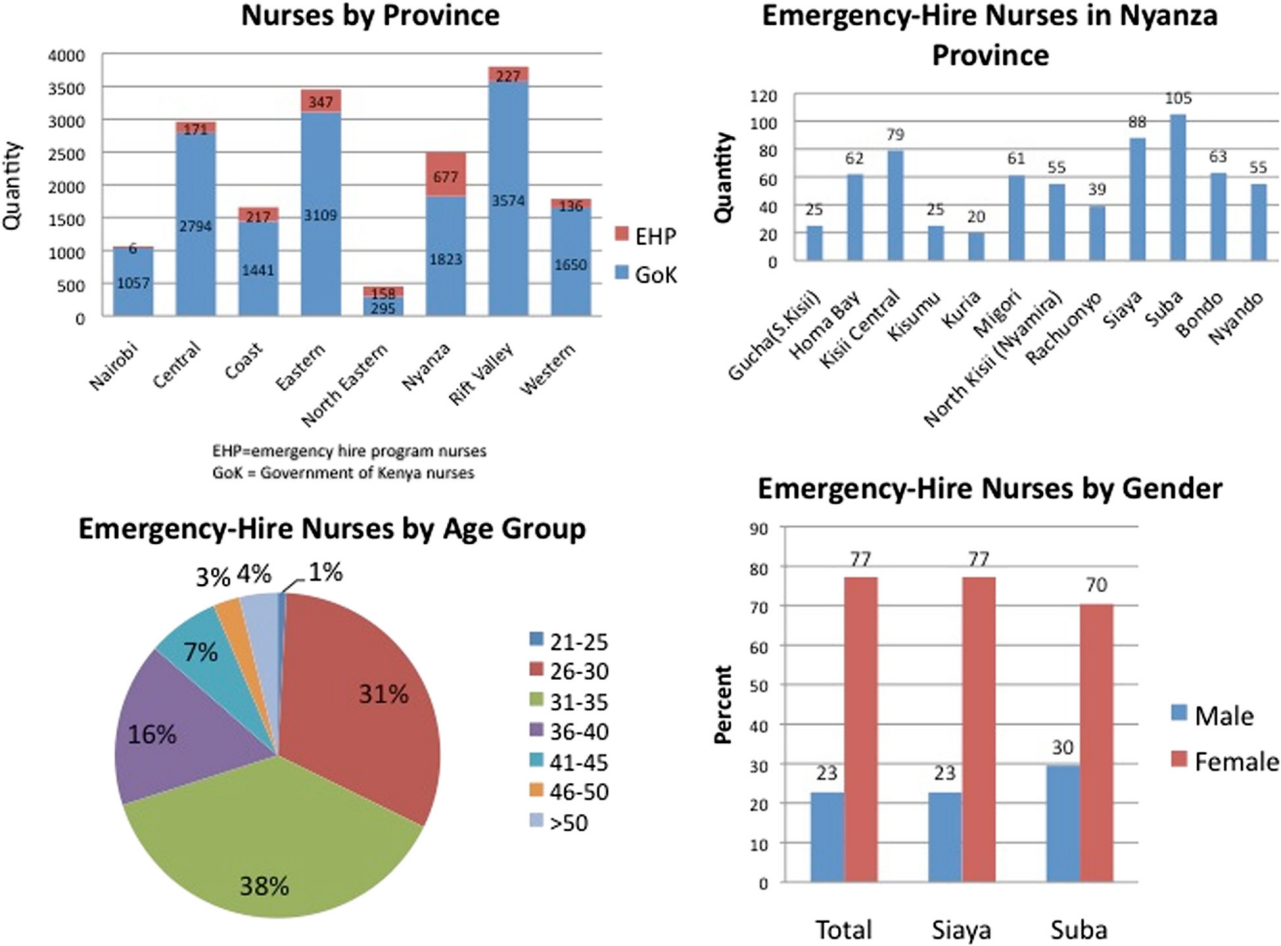
**Characteristics of nurses in Kenya: demographics, July 2010.** EHP = emergency-hire programme nurses. GoK = Government of Kenya nurses.

As of 1 June 2010, there were 17 682 nurses working in GoK facilities of which 15 743 (89%) were permanent MOH employees and 1939 (11%) were hired through the emergency-hire programme (Figure 2). Of the EHP nurses, 677 (35%) nurses were assigned to Nyanza Province, the largest allocation of EHP nurses to any province. Within Nyanza Province, most nurses were within four districts, including Suba (15.5%) followed by Siaya (13%), Kisii (11.7%), and Bondo (9.3%). Most EHP nurses are aged 31–35 (38%) or 26–30 (31%) (Figure 2) and predominately female (77%), including in Suba (70%) and Siaya (77%) Districts.

Health facility data on health service delivery is described in Tables 1 and 2. Individual hospital trends as well as the district trend (thick, black line) can be seen for the six key variables. Data availability improved over time. In Siaya, 32% of 2006 data were obtained, which increased to 85%, 91% and then decreased to 84% in 2007, 2008, and 2009 respectively. Findings were similar in Suba with 28%, 78%, 82%, and 83% of data available from 2006, 2007, 2008, and 2009 respectively.

**Table 2:** Annual trends of aggregate data in Siaya and Suba Districts

**Siaya**	**Suba**
**Total general outpatients**	
	
**Vaginal deliveries**	
	
**Caesarean sections**	
	
**Total antenatal care attendees**	
	
**Antenatal care clients tested for HIV**	
	
**Total deliveries from HIV + women**	
	

*Districts summarized by average overall sites.

†Note differences in scale.

‡Note: Red vertical reference line indicates the year within which nurse staffing was escalated.

Overall, there was a significant increase in the quantity of total general outpatient visits from 2006 to 2007 in Siaya (*P* = 0.01) and Suba (*P* = 0.04) (Table 1); this increase plateaued in 2008. There was a similar significant increase in vaginal deliveries from 2006 to 2007 (Table 1). Caesarean sections also increased significantly in Siaya from 2006 to 2007 (*P* < 0.0001) and from 2007 to 2008 (*P* = 0.02), most notably due to hospital site four (Table 2). Similarly in Suba, caesarean sections increased after 2006 (*P* < 0.0001) with some fluctuation down and back up in 2008 and 2009 respectively (Table 2).

Analysis of ANC attendance showed a significant increase from 2006 to 2007 in Siaya (*P* = 0.02); this was sustained in 2008 but began to drop toward 2006 levels in 2009. Suba similarly had increased ANC attendance after 2006 (*P* < 0.0001). However, this was not sustained as ANC attendance levels dropped in 2008 and 2009 almost back to 2006 levels.

Delivery of HIV services was also assessed. In Siaya, there was a significant increase in ANC clients tested for HIV from 2006 to 2007 (*P* < 0.0001). More notable, there was a significant increase in the number of deliveries to HIV-positive women from 2006 to 2007 (*P* = 0.0004), which continued to increase in 2008 (*P* = 0.05) and in 2009 (*P* = 0.04). Suba similarly had significant increases in ANC client HIV testing and deliveries to HIV-positive women in 2007, 2008, and 2009.

Key informant interviews revealed most government nurses were pleased with the overall service delivery effects of the EHP (common themes in Table 3). With regard to health facility operation, additional nurses were trained in speciality areas, such as maternal-child health, HIV/AIDS, or paediatrics. This ‘departmentalization’ allowed for more focused patient care and decreased burnout among nurses who were previously required to cover multiple departments. Additionally, better staffed facilities were now able to provide emergency services overnight, including obstetric care, thereby limiting the distances women had to travel for labour and delivery, potentially contributing to decreasing the frequency of home births. Key informants also reported that increased staff allowed for more specialized HIV/AIDS services, including testing of patients and their partners, disease management, and education on prevention.

**Table 3:** Common themes from key informant interviews

**Discussion topic**	**Common themes by respondents**
Impact of the emergency-hire programme (EHP) on health-care providers/clinic	Ability to train in speciality areas (e.g. paediatrics, HIV/AIDS, maternal-child health), also referred to as departmentalization
	Decrease in nurse burnout
	Ability to increase emergency services overnight
Impact of the EHP on patients within the community (per clinic staff report)	More patients seen each day
	Decreased waiting times for patients
	Increased access to obstetrical care, particularly overnight deliveries
	Increased patient-provider time to discuss HIV/AIDS management and prevention
Challenges to maintaining adequate staffing levels	Nurse placement far from home village resulting in difficulty adapting to a new, often rural, village and missing husband/children
	Difficulty adapting to a new language/dialect and culture
	Retirement and out-migration were less commonly reported
Barriers to further increasing delivery of health services	More staff needed, despite addition of EHP nurses
	Rainy season
	Rough terrain
	Limited ambulance services to transport critically ill patients to larger hospitals
	Limited availability of medications (except HIV meds) and medical supplies (e.g. gloves, syringes)
Additional factors affecting delivery of health services	Presence of nongovernmental organizations (NGOs) in the region
	Political unrest, particularly at time of elections (2008)
	Intermittent outbreaks (e.g. cholera)
Potential improvements to future EHPs (per clinic staff report)	Placement of nurses in districts/provinces near family
	Increased wages and uniform stipends
	Availability of educational courses
	Enhanced housing options
	Easier process of absorption into the Government nursing force

Although nurse managers agreed the EHP resulted in an overall increase in staffing levels, many commented that one of the challenges was retaining nurses for the duration of his/her three-year contract. Nurses frequently did not want to remain due to difficulty adapting to a new, rural district with rough terrain and lengthy rainy seasons coupled with long distances from family.

In staff discussions, we also focused on environmental factors that may have influenced service delivery beyond staffing levels. Staff reported that the presence of nongovernmental organizations (NGOs) and community health workers likely played a role in promoting deliveries by skilled health professionals. Additionally, staff commented on the intermittent inability to obtain medications, limited ambulance services, and rainy weather as barriers to offering health services.

## Discussion

We present data clearly showing a statistically significant increase in the provision of primary care services from 2006 to 2007, when EHP staff was hired. We found a significant increase in all six service delivery indicators we measured, which is likely related to increased patient visitation, enhanced staffing and more efficient provision of care allowing for more daily encounters. The departmentalization of new nursing staff appeared to be beneficial both quantitatively and qualitatively.

Additional staff also enabled several health facilities to remain operational 24 hours, which was important for overnight obstetrical care. Labouring women no longer had to travel long distances to larger hospitals as local staff were available overnight to perform this service. For more complicated pregnancies requiring caesarean section, larger facilities now had the nursing staff to assist with these procedures; however, this service was impacted by other health systems components, such as, availability of ambulance services and anaesthetics. Our data illustrate that increased staff allowed for more facility-based deliveries, both vaginal and caesarean. Although one would anticipate that the number of pregnant women would not have changed significantly, it is likely that mothers who previously chose to deliver at home with lay or untrained midwives now travelled to the clinic due to increased accessibility. This trend may have been enhanced by local NGOs advocating for health-care facility-based deliveries aimed at decreasing infant mortality related to home births. Our data suggests that increased ANC attendance may have also played a role. Studies have shown that access to ANC early in pregnancy results in increased skilled professional birthing assistance, which decreases infant and maternal mortality [17].

With additional nurses, health centres were also better equipped to assess pregnant mothers for HIV/AIDS and provide services to limit mother-to-child transmission to infants. Despite widespread education about HIV/AIDS prevention, we found increasing numbers of HIV-positive pregnant women. This is likely a combination of continued HIV transmission and increased testing by health-care providers. There was also a significant increase in facility-based deliveries to HIV-positive women. Due to a combination of increased facility-based deliveries and active provision of antiretrovirals to labouring mothers and their newborns, it is likely that many infant HIV infections may have been prevented. Indicators show more than 660 000 HIV-positive women in PEPFAR countries were supported with HIV prophylaxis resulting in approximately 200 000 infants born HIV-free in 2011 [18].

Although our results demonstrate multiple improvements in service provision immediately following nurse placement, we did not routinely see continued increases in outpatient visits, deliveries, and ANC attendance one and two years following enhanced nurse staffing levels. This is most likely related to saturation of services that could not be supplied without an additional increase in nurse staffing. Despite a lack of continuing rise in services, we routinely found a gain in services from 2006 to 2007, immediately following EHP placement, that were sustained in 2008 and 2009. Sustaining these efforts beyond the third year will likely require new investments in health workforce such as improved incentives (e.g. increased wages, availability of educational courses, uniform stipends), enhanced housing options, and the opportunity for nurses to choose their rural location [19,20]. Also, the recruitment of new staff cannot be a one-time effort since staff will continue to retire, transfer, and migrate on an annual basis. Without continued maintenance of existing workforce levels, ideally with additional staffing increases, it will be impossible for health services and interventions to be further enhanced.

A follow-up review of current staffing levels in May 2013 indicates a total of 18 625 nurses working in the public sector, which is a 5% increase from 17 682. Among the subset of nurses hired by donors with absorption agreement, 94% of nurses were absorbed into the public sector by February 2010 [15]. Data from other donors without established GoK agreements for absorption do not demonstrate GoK inclusion following the end of their contract (n = 159).

### Strengths and limitations

There are several strengths and limitations of this evaluation. Although we have accurate information on the number of EHP nurses deployed, the involvement of various donor groups participating in the hiring process makes it difficult to determine exact starting dates for each nurse. Generally, we assign a baseline time period (2006), scale-up period (2007), and post scale-up period (2008 and 2009); however, due to variations in hiring practices, these may not be strictly accurate. Related, individual facilities did not have records available with the exact quantity of nurses working at any given time, such as in 2006; however, as described, we have accurate national data describing where EHP nurses were initially placed.

We present aggregate data describing information from multiple health-care facilities in two districts. This increases the risk of ecological fallacy. While there are clear trends, individual facilities may have varying significance depending on the variable under analysis. For example, during a period of Kenyan national election violence—greatest in early 2008—there were sharp declines in patient visits. Following this nadir, there was a notable rise in outpatient visits. This was noted in Siaya, which is geographically closer to Kisumu, a site of significant violence. In addition to staffing levels, there were other factors reported by staff that influenced service delivery; thus, the increase in service delivery may not be solely related to increased staffing, but a combination of efforts. Some of these factors were obtained through informal discussion, although these discussions were limited by the convenience of available staff and variable levels of training of staff and his/her involvement in health service delivery.

This assessment was designed as a pilot evaluation; therefore, a small sample size was used without controls sites (e.g. sites where no EHP nurses were placed). Despite this, we were able to use pre-intervention data from selected sites which serve as a point of comparison. Additionally, this evaluation successfully assessed the feasibility of performing a small, low-cost pilot study. This is the first field-level evaluation in Kenya examining the linkage between HRIS and HMIS or the EHP’s effect on local service delivery. As such, it has helped identify the many successes and challenges to alleviating staffing shortages and document the impact of HRH interventions in Kenya.

## Conclusions

While this evaluation focused on Kenya’s EHP and the effects on health service delivery, as our qualitative results illustrate, other factors also positively and negatively affect health services, including weather, emergency services, political turmoil, and working conditions. Each of these individual components provides potential topics for further investigation. Similarly, as this evaluation did not aim to comment on the EHP structure or implementation, additional studies should examine other models targeting workforce supply, including pre-service training and workforce incentives. Significantly, this evaluation examined previously uncollected data and through discussions with health-care providers helped to identify multiple issues impacting delivery of primary care services. As a result, these findings should encourage further research to better assess the long-term sustainability of this intervention. In performing this research, the authors had confidence in the quality of HRIS data, although documentation of HMIS data was variable depending on the site. The completeness of HMIS data did appear to improve over the years studied with guidance and additional record-keeping training from the MOH. Additionally, to enhance the power of future studies, it may be beneficial to utilize sites where no EHP nurses have been placed to serve as control sites.

Beyond the assessment of EHP impact on health service delivery, this evaluation also helped to determine the feasibility of performing a small, low-cost pilot study in a rural area. This study stressed the importance of strong collaboration with Kenyan partners, both at the Ministry of Health and community levels. The involvement of local health-care staff was essential to performing this evaluation. This lesson of fostering collaboration should be a basis for future work.

## Competing interests

All authors declare: no support from any organization for the submitted work; no financial relationships with any organizations that might have an interest in the submitted work. The authors declare that they have no competing interests.

## Authors’ contributions

SV was involved in project conception, protocol development, data collection, data analysis, manuscript development, and manuscript review. PR was involved in project conception, protocol development, manuscript development, and manuscript review. FK was involved in manuscript review. RW was involved in protocol development, data collection, and manuscript review. PW was involved in protocol development, data collection, and manuscript review. JS was involved in data analysis and manuscript review. RK was involved in data analysis and manuscript review. MF was involved in project conception, protocol development, and manuscript review. MO was involved in protocol development and manuscript review. AW was involved in manuscript review. CR was involved in manuscript review. MR was involved in project conception, protocol development, manuscript development, and manuscript review. All authors read and approved the final manuscript.

## Supplementary Material

Additional file 1 **(a) Government of Kenya Health Management Information System forms. (b)** Government of Kenya Health Management Information System forms. Click here for file

Additional file 2 Interview guide for evaluation of the impact of the Kenya Emergency-Hire Nursing Programme on the delivery of health services. Click here for file
