# Supplementary material for: Kenya’s emergency-hire nursing programme: a pilot evaluation of health service delivery in two districts
Source: Hum Resour Health. 2014 Mar 17;12:16. doi: 10.1186/1478-4491-12-16 (PMC4003900; doi:10.1186/1478-4491-12-16)
Supplement: Additional file 1 — (a) Government of Kenya Health Management Information System forms. (b) Government of Kenya Health Management Information System forms. [file 1478-4491-12-16-S1.doc]

Additional file 1a: GoK HMIS forms

Additional file 1b: GoK HMIS forms
