# Supplementary material for: Kenya’s emergency-hire nursing programme: a pilot evaluation of health service delivery in two districts
Source: Hum Resour Health. 2014 Mar 17;12:16. doi: 10.1186/1478-4491-12-16 (PMC4003900; doi:10.1186/1478-4491-12-16)
Supplement: Additional file 2 — Interview guide for evaluation of the impact of the Kenya Emergency-Hire Nursing Programme on the delivery of health services. [file 1478-4491-12-16-S2.doc]

Additional file 2:

**Interview Guide**

**Interview Guide for**

**Evaluation of the Impact of Kenya Emergency-Hire Nursing Program**

**on the Delivery of Health Services**

Name of Interviewee: ___________________________________________________________

Position/Role: ___________________________________________________________

Dates of Tenure at Facility: _______________________________________________________

Facility Location: ___________________________________________________________

Date: _________________________________________________ Time: __________________

**Questions for discussion**

Has there been a change in the levels of nurse staffing at this facility since 2004?

If so, how has this change impacted your delivery of health services?

How would you describe the delivery of health services before and after these additional nurses were hired?

Has it increased the quantity of services offered? Examples?

Has it altered the quality of services offered? Examples?

Is it purely the changes in nurse staffing levels or are there other factors contributing to changes in the quantity/quality of health services? If other factors, what are they?

Were the changes in nurse staffing levels a result of Kenya’s emergency-hire program (EHP) or were there non-EHP hires as well? Can you give an estimate of how many nurses were hired in each category (EHP vs non-EHP) over the past 5-10 years?

When EHP nurses were hired, were existing nurses shifted around to other facilities or did they continue to work at the same facility, resulting in an overall increase in staffing levels?

Has this facility experienced any loss of nurses in the past 1-2 years (deaths vs out-migration vs. transfer to another facility)? Do you think this has affected health service delivery?

Of the EHP nurses added to your facility, what level of training did they have (enrolled vs. registered vs. BScN)?

Have there been any major or newly implemented health programs or health campaigns that have directly impacted this health facility over the last five years that may have affected the quantity of health services delivered?

Has there been any enhancement in road construction locally that may have impacted delivery of health services?

Have you had any problems with absenteeism of nurses or other staff in your facility?

Can you estimate approximately how many patients are seen in this health facility daily?

Have there been any barriers to increasing the quantity/quality of health service delivery?

Have there been any problems with the availability of commodities, such as drugs or medical devices?

Do you have any other comments about the emergency-hire program or your facility’s current nurse staffing level?
